# Randomized clinical trial of the timing it right stroke family support program: research protocol

**DOI:** 10.1186/1472-6963-14-18

**Published:** 2014-01-17

**Authors:** Jill I Cameron, Gary Naglie, Monique A M Gignac, Mark Bayley, Grace Warner, Theresa Green, Anna Czerwonka, Maria Huijbregts, Frank L Silver, Steve J Phillips, Angela M Cheung

**Affiliations:** 1 Department of Occupational Science and Occupational Therapy, University of Toronto, 160-500 University Ave, Toronto, Ontario M5G 1V7, Canada; 2 UHN Toronto Rehabilitation Institute, Toronto, Canada; 3 Department of Medicine and Rotman Research Institute, Baycrest Health Sciences, 3560 Bathurst Street, Toronto, Ontario M6A 2E1, Canada; 4 Research Department, UHN Toronto Rehabilitation Institute, Toronto, Canada; 5 Department of Medicine and Institute of Health Policy, Management and Evaluation, University of Toronto, Toronto, Canada; 6 UHN Toronto Western Research Institute, 399 Bathurst Street, Main Pavilion, 10-328, Toronto, ON ON M5T 2S8, Canada; 7 Dalla Lana School of Public Health, University of Toronto, Toronto, Canada; 8 UHN Toronto Rehabilitation Institute, University Centre, University of Toronto, 550 University Avenue, Toronto, Ontario M5G 2A2, Canada; 9 Department of Medicine, University of Toronto, Toronto Canada; 10 School of Occupational Therapy, Dalhousie University, 5689 University Ave, Halifax, Nova Scotia B3H 3J5, Canada; 11 Faculty of Nursing, University of Calgary, 2500 University Dr NW, Calgary AB T2N 1N4, Canada; 12 Family Service Toronto, 355 Church Street, Toronto, Ontario M5B 1Z8, Canada; 13 Department of Physical Therapy, University of Toronto, Baycrest Health Sciences, Toronto, Canada; 14 UHN Toronto Western Hospital, 399 Bathurst Street, Toronto, ON M5T 2S8, Canada; 15 Department of Medicine, Division of Neurology, Dalhousie University/QEII Health Sciences Centre, 1796 Summer Street, Halifax, Nova Scotia B3H 3A7, Canada; 16 UHN Toronto General Hospital, 200 Elizabeth Street, 7 Eaton North - room 221, Toronto, Ontario M5G 2C4, Canada; 17 Department of Medicine, University of Toronto, Toronto, Canada

**Keywords:** Stroke, Caregiver, Mixed methods, Randomized controlled trial, Social support, Education, Longitudinal

## Abstract

**Background:**

Family caregivers provide invaluable support to stroke survivors during their recovery, rehabilitation, and community re-integration. Unfortunately, it is not standard clinical practice to prepare and support caregivers in this role and, as a result, many experience stress and poor health that can compromise stroke survivor recovery and threaten the sustainability of keeping the stroke survivor at home. We developed the Timing it Right Stroke Family Support Program (TIRSFSP) to guide the timing of delivering specific types of education and support to meet caregivers’ evolving needs. The objective of this multi-site randomized controlled trial is to determine if delivering the TIRSFSP across the stroke care continuum improves caregivers’ sense of being supported and emotional well-being.

**Methods/design:**

Our multi-site single-blinded randomized controlled trial will recruit 300 family caregivers of stroke survivors from urban and rural acute care hospitals. After completing a baseline assessment, participants will be randomly allocated to one of three groups: 1) TIRSFSP guided by a stroke support person (health care professional with stroke care experience), delivered in-person during acute care and by telephone for approximately the first six to 12 months post-stroke, 2) caregiver self-directed TIRSFSP with an initial introduction to the program by a stroke support person, or 3) standard care receiving the educational resource “Let’s Talk about Stroke” prepared by the Heart and Stroke Foundation. Participants will complete three follow-up quantitative assessments 3, 6, and 12-months post-stroke. These include assessments of depression, social support, psychological well-being, stroke knowledge, mastery (sense of control over life), caregiving assistance provided, caregiving impact on everyday life, and indicators of stroke severity and disability. Qualitative methods will also be used to obtain information about caregivers’ experiences with the education and support received and the impact on caregivers’ perception of being supported and emotional well-being.

**Discussion:**

This research will determine if the TIRSFSP benefits family caregivers by improving their perception of being supported and emotional well-being. If proven effective, it could be recommended as a model of stroke family education and support that meets the Canadian Stroke Best Practice Guideline recommendation for providing timely education and support to families through transitions.

**Trial registration:**

ClinicalTrials.gov: NCT00958607.

## Background

Stroke is a complex medical condition and, as a result, stroke survivors utilize many elements of the health care system including acute, rehabilitation, and community care services. In Canada, an estimated 58% of stroke survivors go directly home after acute care, 19% go to inpatient rehabilitation before going home, and 10% are admitted to long-term care [1]. As many as 50% of stroke survivors returning to the community have difficulties performing every day activities, including bathing, walking short distances, negotiating stairs, housework, meal preparation and traveling [2]. Family caregivers assist stroke survivors with daily activities and navigating health care services [3]. Currently, there is no standard clinical practice to prepare family members for this caregiving role. As a result, caregivers often experience stress and poor mental and physical health [4-6] that can contribute to poor rehabilitation outcomes for stroke survivors [7] or threaten the sustainability of care at home [8,9]. At the same time, health care delivery systems have been criticized for reducing hospital lengths-of-stay [10], for limiting availability of community services [11], and for lacking continuity across services [12]. This increases the demand placed upon family caregivers as they support individuals with considerable disability across fragmented systems of care with very limited support from community resources. To date, programs have been developed to provide stroke-specific information and caregiver training and counseling, but these have had only a small impact on improving caregiver well-being [13]. One possible explanation, for this for which there is emerging evidence, is that these programs do not consider caregivers’ changing needs across the care continuum [14,15]. We developed the Timing it Right (TIR) framework that aims to guide the appropriate timing of specific types of education and support to meet the evolving needs of caregivers [16].

### “Timing it right” (TIR) framework

**Figure 1:**
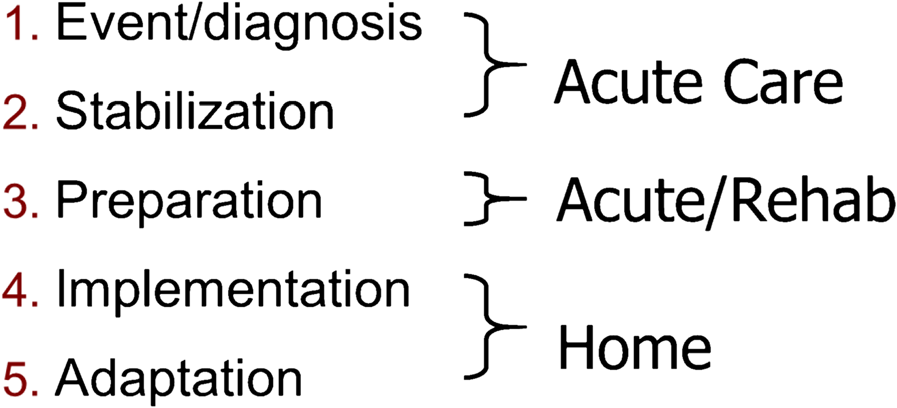
Timing it right framework (Cameron and Gignac, 2008).

The “Timing it Right” (TIR) framework promotes an organized approach to developing and evaluating interventions aimed at meeting family caregivers’ changing needs for support [16]. The TIR framework emphasizes family caregivers’ unique support needs for each of five phases as described in Figure 1. The event/diagnosis phase concerns the time surrounding the stroke event and emergency care. This phase ends when the person’s medical condition has stabilized, at which point caregivers enter the second phase, stabilization. The preparation phase occurs when the stroke survivor is preparing for discharge home from inpatient acute or rehabilitation care. The implementation phase concerns the first few months after the stroke survivor returns home and they are adjusting to living in the community. The adaptation phase occurs once they are comfortable living in the home environment and their emphasis shifts to community re-integration.

The premise of the framework is that careful attention to phase-specific needs for concrete information and strategies will enhance family caregiver preparedness, ease their transitions across the care continuum, and decrease the occurrence of negative outcomes [16] (e.g., burden, depressive symptoms, and other health issues). Ultimately, there will also be enhanced care of the person with stroke. Interventions developed according to the TIR framework target the support provided to caregivers according to their phase in the TIR framework.

Existing caregiver interventions have drawn substantially from research on social support and aim to provide different elements of support including emotional (e.g., providing comfort, listening to problems [17,18]), instrumental (e.g., providing training in problem-solving [19,20]), informational (e.g., providing information about illness and services [18,21-23]), and appraisal support (e.g., providing feedback about their caregiving activities [24]). Therefore, we have placed the TIR framework in this context of social support. Previous research has demonstrated that family caregivers who can draw upon social support will experience better mental health outcomes [25-27]. In addition, support is most beneficial if it is closely matched to an individual’s current needs [28]. This reinforces the need to more closely examine caregivers’ support needs over time and provide support when and where they need it.

### Previous caregiver intervention research

To date, some interventions have been developed to promote caregivers’ adaptation to their caregiving role (see reviews [13,29]). These interventions often provide information about the illness and treatment [18,21,22,30] or about community services [23]. Some provide caregiver counselling [17,18] or training to assist with solving caregiving problems [19,20,31]. In addition, others have proposed changes to the delivery of acute care and inpatient rehabilitation (e.g., early supported discharge [32], integrated care pathways [33]) that may also benefit family caregivers. Some investigators have studied caregiver interventions that cross a portion of the care continuum, most frequently from acute care to the home. These interventions typically begin when the patient is still in acute care and consist of home visits and/or telephone support to continue the intervention in the community [18,20]. Some interventions use trained nurses [20] or family support organizers [18] to deliver the intervention and have demonstrated some benefit in caregiver outcomes (e.g., constant social support). Similarly, telephone support provided by nurse educators for family caregivers has been associated with small improvements in caregiver problem-solving skills, mental health, perception of preparedness, vitality, and social functioning [19,34,35]. Reviews suggest that these interventions result in small improvements in caregiver burden, emotional distress, psychological well-being, and quality of life [29]. Larger improvements in outcomes are observed with interventions that are tailored to individual caregivers’ needs and are psychotherapeutic, psycho-educational, or multi-component in nature [29]. To date, none of these interventions have specifically considered: 1) the appropriate timing of caregiver education and support; and 2) changes in caregivers’ needs as they provide support to stroke survivors moving across the care continuum. Providing caregivers with the support they need when they need it may be of additional benefit.

The appropriate model for intervention delivery across the care continuum is not yet known. In our qualitative research, caregivers indicated a need for one key individual to be available to them to answer questions and provide support as needed while the stroke survivor was in the hospital and after they had returned home [15]. The stroke support programs discussed above used trained nurses [20] or family support organizers [18] to deliver the intervention in person and by telephone [19,34,35]. Other models have focused on providing integrated service delivery to enhance the coordination of care as patients move across the care continuum [12,36-38]. These commonly use a system where one individual (e.g., a case manager [36]) organizes and facilitates a patient’s access to services across the health care system. The common thread of this research suggests we need to have one key individual provide support across care environments.

In addition, some recent research has highlighted the benefits of preparing patients and their family members to self-manage their transitions across care environments [39,40]. By providing family members with education and guidance, the self-management approach can be extended to enable family members to self-direct their learning and support needs across care environments. This second alternative for intervention delivery may be a cost-effective way for caregivers to obtain the information, training and support that they require when they require it.

## Research objectives and hypotheses

The objective of this multi-site mixed methodology single-blind randomized controlled trial is to determine if the TIRSFSP delivered across the care continuum contributes to positive caregiver outcomes. Since this program targets family caregiver support, the primary outcomes of the intervention will be caregivers’ perception of being supported in their caregiving role and improvements in caregiver mental health outcomes (e.g., less depression and more psychological well-being). To determine the impact of the intervention on caregiver outcomes, we will compare two modes of intervention delivery with standard care: 1) repeated contact in person and by telephone with a stroke support person (health care professional with stroke care experience, intervention arm 1); and 2) a self-directed program by the caregiver (intervention arm 2). The specific research hypotheses are the following:

### Hypotheses

1. Family caregivers receiving the TIRSFSP delivered by a stroke support person and the self-directed TIRSFSP will report more perceived support and better mental health than the standard care group.

2. Family caregivers receiving the TIRSFSP delivered by a stroke support person will report more perceived support and better mental health compared to those receiving the self-directed TIRSFSP and standard care.

## Methods

### Trial design

We will conduct a convergent parallel mixed methodology [41], longitudinal, multi-site, single-blind randomized controlled trial. As a convergent parallel mixed methodology design, the quantitative and qualitative portions of the study will provide different but complementary perspectives on caregivers’ experiences with support received [41]. Recruitment will take place in 7 acute care hospitals from across Canada. Caregivers will be recruited during the patients’ acute care hospital admission. They will complete baseline assessments prior to randomization and follow-up assessments at 3, 6, and 12-month’s post stroke. All caregivers will provide written informed consent. The research protocol has been approved by institutional research ethics boards at all participating hospitals, including the following: University Health Network Research Ethics Board, Capital Health Research Ethics Board, Ottawa Hospital Research Ethics Board, University of Toronto Office of Research Ethics, University of Calgary Faculty of Medicine Office of Medical Bioethics Conjoint Health Research Ethics Board, Pembroke Regional Hospital Research Ethics Committee, Thunder Bay Regional Health Sciences Centre Research Ethics Board, Lakeridge Health Research Ethics Board, Queen’s University Health Sciences & Affiliated Teaching Hospitals Research Ethics Board, Health PEI Research Ethics Board, Royal Victoria Hospital Research Ethics Board, and Cape Breton District Health Authority Research Ethics Board.

### Participants

Family caregivers are defined as the person primarily responsible for providing and/or coordinating stroke survivor care in the community without financial compensation. If during acute care there appears to be more than one family caregiver, we will invite the family member who, in discussion with the family, is likely to be primarily responsible for providing and/or coordinating care in the home.

#### Inclusion criteria

Participants will be included if they are able to read and speak English and will be caring for a person who is either receiving care for their first hospitalization for an ischemic or hemorrhagic stroke or whose previous stroke was mild and they did not require admission to inpatient or outpatient rehabilitation care. The stroke survivor’s anticipated ultimate destination after discharge is a private residence or apartment building. Stroke survivors must exhibit at least minimal disability (i.e., referred to an occupational therapist, physical therapist, or speech language pathologist during acute care). Stroke survivors may be admitted to short or long-duration inpatient (maximum duration of 6 months) or outpatient rehabilitation or return directly home. During inpatient rehabilitation, there will be stroke survivors who do not reach their rehabilitation goals and will, therefore, not be able to return home. When this occurs, we will exclude their caregivers from the study.

#### Exclusion criteria

We will exclude caregivers of patients who are terminally ill, discharged to alternative levels of care, or discharged to long-term care or assisted retirement residences.

### Interventions

Participants in intervention arms 1 and 2 will receive the TIRSFSP Guide. This educational resource contains an introductory chapter, one chapter for each phase in the TIR framework, and a concluding chapter containing lists of local resources available to the patient and caregiver (see Table 1). The content of each chapter is based on the TIR framework [16] and caregivers descriptions of their phase-specific experiences and needs obtained during a previous qualitative study [15]. We leveraged existing educational and support resources, created new resources, and organized them according to the phases of the TIR framework. Each chapter provides emotional support by describing how some caregivers may feel emotionally during each phase [15]. Each chapter also provides a “who to talk to for help” section to guide caregivers as to who they should speak with for certain areas of concern (e.g., speech language pathologist to learn more about communicating with a stroke survivor with aphasia). The introduction to the guide instructs caregivers to “self-manage” their support needs [42,43]. It provides caregivers with strategies for eliciting help and support from family and friends, communicating effectively with health care professionals, obtaining community services, and succeeding at caregiving. Caregivers will be instructed to review the information in each chapter as it becomes relevant to their current situation (i.e., as they move through the TIR phases). Caregivers can review the guide as often as they like.

1. Stroke Support Person TIRSFSP: The intervention will be delivered by a stroke support person in-person during the acute care phase and then by monthly telephone calls for approximately six months after the stroke survivor is discharge home. The stroke support person is a health care professional involved in the coordination of stroke services and provision of education and support (e.g., occupational therapist, social worker, nurse educator, case manager). During each session, the stroke support person will ask caregivers how things have been going, how they are doing, and what their needs are so they can guide them through appropriate sections of the TIRSFSP educational resource and help them obtain the supports they currently need. The stroke support person will encourage, using self-management principles, caregivers to obtain the needed supports from the appropriate health care professional (e.g., training in caregiving activities from an occupational therapist) or appropriate community resources. They will give the caregivers a copy of the TIRSFSP guide as a resource. The support person will encourage the caregiver to contact them between regular appointments if they have any specific questions. Our pilot study indicated that the first meeting is the longest and takes approximately 60 minutes and subsequent meetings are 10–30 minutes each [44]. The intervention content and duration, including time spent during each session and number of sessions, is tailored to individual caregivers’ experiences and needs.

2. Self-Directed TIRSFSP: In the second arm of this trial, family caregivers will self-direct their use of the TIRSFSP guide. The stroke support person will meet with the caregiver once during the acute stroke phase to instruct them on the use of the guide. They will provide an overview of the self-management principles and encourage them to manage their support needs.

3. Standard Care: Current standard care in the stroke centres of the Ontario Stroke System is for stroke survivors and/or family members to receive a copy of the Heart and Stroke Foundation’s “Let’s Talk about Stroke” educational resource. This resource provides general information that is not specific to phase of recovery, to educate stroke families about what a stroke is, treatment options, secondary prevention, and impact on stroke survivors’ health and well-being. The research assistant will ensure that all caregivers recruited into the trial receive a copy of “Let’s Talk about Stroke” with a brief introduction to its contents.

**Table 1:** Contents of the timing it right stroke family support program guide

**Chapter**	**Title**
1.	Introduction to the program
2.	My family member has had a stroke
3.	My family member’s condition has stabilized
4.	My family member is preparing to go home
5.	My family member has just returned home
6.	Adapting to life in the community
7.	Additional resources

### Data collection

Three sources of data will be collected in the form of structured quantitative measurement instruments, qualitative interviews, and stroke support person journals.

Structured Quantitative Measurement Instruments: Caregiver outcomes are the focus of this study. The research assistant in each region will obtain the baseline and follow-up data at 3, 6, and 12-months post-stroke. All measurement instruments have been used previously in stroke caregiving research and demonstrate good psychometric properties [45-47]. In the pilot study, administration of these quantitative measures took approximately 30–45 minutes [44]. All data will be entered into the EmPower web-based system that will support and manage the data this clinical trial [48]. Caregivers will be able to complete mailed surveys or enter their responses directly into EmPower.

The primary outcome, caregiver’s perceived social support, will be assessed by the Medical Outcomes Study Social Support Scale [49]. Positive and negative mental health outcomes, will be assessed by the Positive Affect Scale [50] and the Centre for Epidemiological Studies Depression Scale [51], respectively. Caregivers’ participation in valued activities, will be assessed by the Caregiving Impact Scale [45,52]. The level of assistance provided to the stroke survivor in terms of activities of daily living, instrumental activities and medical care will be assessed by the Caregiving Assistance Scale [45,52]. Caregivers’ stroke knowledge concerning warning signs, causes, consequences, treatment options, rehabilitation, and secondary prevention will be assessed by the Stroke Knowledge Test [53]. Caregivers’ sense of control over life will be assessed by Pearlin’s Mastery Scale [54]. Personal development as a result of providing care will be assessed by Pearlin’s Personal Gain Scale [55]. To facilitate an economic analysis related to the intervention, we will also collect Health Resource Utilization data (adapted from Brown, 1990 [56]. Sociodemographic characteristics of the caregiver will also be assessed. Stroke patients’ charts will be reviewed to obtain the following information: stroke severity (Canadian Neurological Scale [57] completed during the first week of hospital admission), functional status as measured by the Barthel Index [58], stroke type and location, and date of stroke.

#### Qualitative interviews

A sub-sample of 36 (12 per intervention arm) caregivers will participate in a qualitative interview after the completion of the final follow-up assessment. Research assistants with training in qualitative interviewing will conduct all the interviews by telephone. Telephone interviews have been shown to provide qualitative data of the same quality and quantity as in-person interviews [59]. We will use framework methodology to describe caregivers’ experiences and needs during each phase of the TIR framework and how the intervention arm that they received did or did not meet their needs [60,61]. Based upon our pilot study, we estimate the qualitative interviews will take approximately 45–60 minutes [44].

#### Stroke support person journals

For each site, the stroke support person will complete a journal to record the details of the intervention provided to participants. Specifically, for each contact with each caregiver, they will record the duration of the contact, topics discussed, and resources provided. This information will help us to understand more fully the role that the stroke support person fills and the time commitment to fill this role.

### Sample size

**Figure 2:**
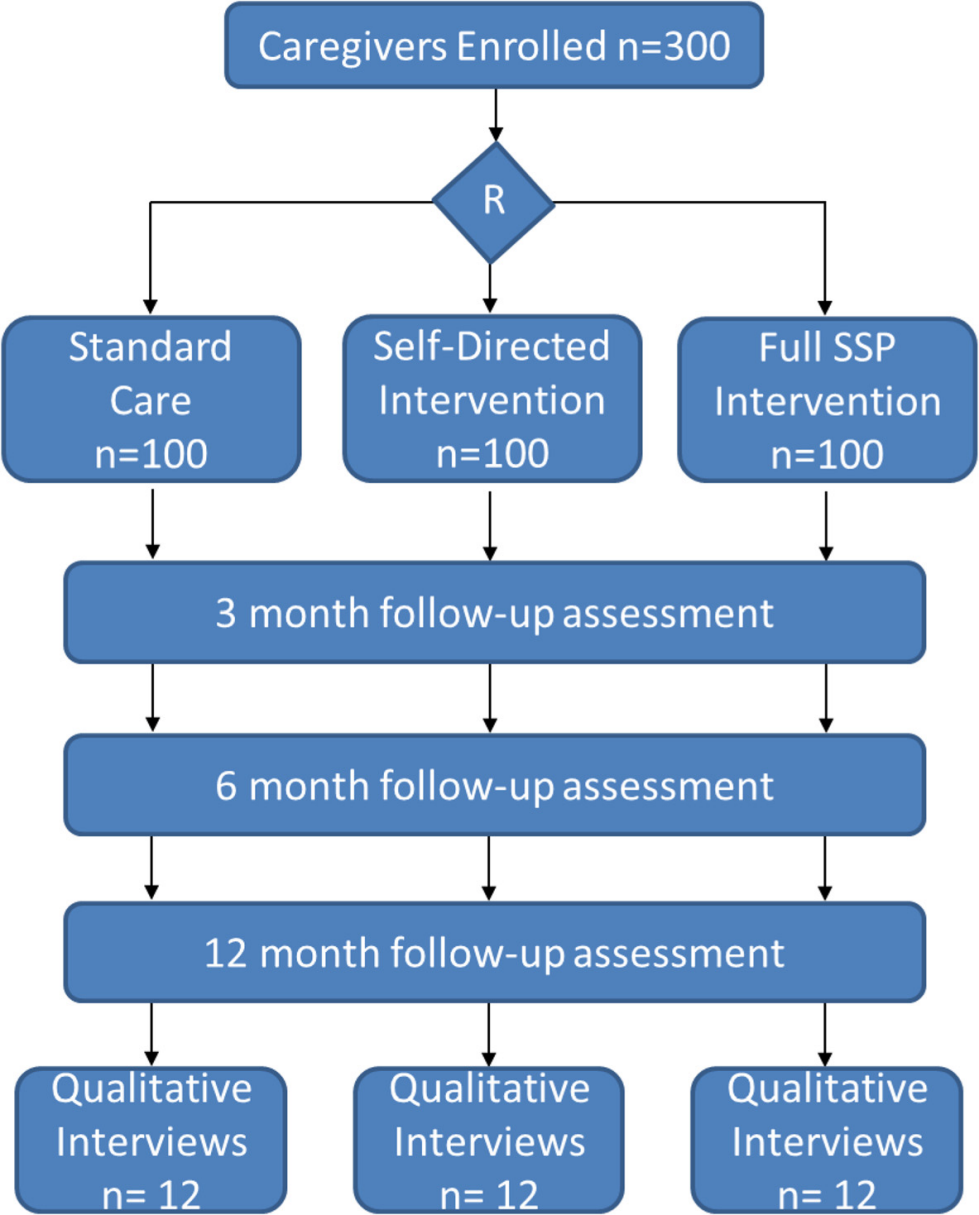
Anticipated CONSORT diagram including follow-up protocol and qualitative data collection.

The target sample size is 300 family caregivers (see Figure 2). With a sample of 100 participants per group we will be able to observe a medium sized difference between three treatment groups in our primary outcome, caregiver’s perceived social support (Social Support Survey, mean 73.3, SD 20.8) (80% power, P < .05 significance) [62]. The estimated sample size remained the same when calculated using the Center for Epidemiological Studies - Depression Score and the Positive Affect Scale as outcome measures (described above). Our sample will include caregivers from urban (n = 240) and rural (n = 60) environments to be representative of the Canadian population (approximately 80% urban and 20% rural). We are using Statistics Canada’s rural definition “A community (generally a municipality in the west and a township in the east) is defined as “rural” if its population density is less than 150 people per square kilometer”.

A sub-set of 36 family caregivers will participate in the qualitative portion of the study. Based on our previous qualitative study with stroke family caregivers, we are confident that this number will ensure saturation of research themes [15,63]. Caregivers will be purposively sampled to reflect the intervention groups (i.e., 12 participants per research group) [64].

### Randomization

Research assistants responsible for recruiting participants into the study will enter baseline data into EmPower and EmPower will use a random number generator to place participants into one of the 3 treatment arms: A) TIRSFSP delivered by a stroke support person, B) TIRSFSP self-directed by the caregiver, and C) standard care. EmPower will email the stroke support person to inform them when a participant has been randomized. We will use stratified randomization to ensure an equal distribution of participants across intervention arms within each site.

### Blinding

The research assistant who collects the baseline and follow-up data will be blind to group assignment.

### Statistical methods

#### Analysis of quantitative data

We will analyze the data on the principle of intention to treat. Hierarchical linear modeling for longitudinal data will examine changes in caregiver outcomes over the follow-up period and examine differences between study groups for each outcome [65]. We will use mixed effects modeling as it accounts for the underlying heterogeneity between and within participants (i.e., intercepts and slopes are allowed to vary across participants). This approach will also allow us to identify differences in rates of change (slopes) in the dependent variables between study groups (e.g., participants in the intervention group with a support person may have earlier improvements in stroke-related knowledge). It will also allow us to control for confounding variables that can also influence caregiver outcomes (e.g., stroke severity). Because intercepts and slopes are computed for each participant, caregivers who miss a follow-up assessment or drop out of the study are still included in the data analyses.

#### Analysis of qualitative data

The in-depth qualitative interviews will be analyzed using the 5 stages of framework analysis: 1) familiarizing by listening to the interviews and reviewing the transcripts; 2) selecting a thematic framework (e.g. TIR model); 3) indexing or coding the data according to the framework; 4) charting the data on the framework, and 5) interpretation [60,61]. This approach will allow examination of caregivers’ changing experiences and needs across the recovery trajectory and determine the extent to which their arm of the intervention met their needs. With this number of qualitative interviews (e.g., 12 participants per intervention arm) we will be able to compare and contrast caregivers’ experiences and needs across intervention arms. Researcher bias is a common threat to the trustworthiness of qualitative data analysis. To minimize this threat we will use the following strategies as recommended by McReynolds [64]: 1) we will maintain an audit trail by keeping record of all data, analysis procedures, and analysis notes; 2) multiple researchers will contribute to the data analysis and theme generation; and 3) we will carefully examine discrepant data (i.e., data that does not support the researchers preliminary conclusions) [64]. We will use NVivo 10 qualitative software to organize the coding process [66].

#### Analysis of stroke support persons’ journals

The stroke support persons’ journals for each phase will be reviewed to determine: 1) the amount of time the stroke support person spends with each caregiver, 2) the topics discussed, and 3) the resources provided. This information will be summarized for each site’s stroke support person and then averaged across sites. The topics and resources provided will be synthesized using conventional content analysis [67,68]. Specifically, for each phase of the intervention, we will count the number of times each topic is discussed and each resource is provided to identify the key topics and resources for each phase of the intervention.

#### Combining findings across different methodologies

Each of these analyses will help us to understand if the intervention improves caregiver outcomes (quantitative analysis), how it does this (qualitative analysis), and how it is delivered (stroke support person journal). This information can be used to inform health care service delivery policy makers about the benefits of the TIR Stroke Family Support Program.

## Discussion

Family caregivers play a central role supporting stroke survivors as they transition from acute care, through rehabilitation, and return to community living. With no standard clinical practice to prepare and support these individuals in the caregiving role, many experience stress and poor mental health outcomes [4-6]. This can compromise the quality of care provided to the patient [7] as well as jeopardize caregivers’ abilities to keep the stroke survivor at home [8,9]. Interventions have been developed and tested to support family caregivers, but have only been able to demonstrate small improvements in caregiver well-being [29]. More recently, Greenwood suggested future research should consider caregivers’ changing needs for support over time [14]. Consistent with the Timing it Right framework, caregivers may have support needs that are specific to the stroke survivor’s place in their recovery trajectory [16]. Our research to date suggests caregivers’ support needs are changing over time and the individuals they prefer to receive support from are also changing [15]. Building upon this information, we have created and pilot tested the Timing it Right Stroke Family Support Program with 30 family caregivers [44]. Preliminary qualitative findings suggest the intervention is meeting the needs of those caregivers who have limited experience with stroke, difficulty obtaining information, and difficulty navigating the health care system [44]. Therefore, improving the timing with which caregiver support is provided may address caregivers’ changing needs as stroke survivors transition across care environments and may result in caregivers being better prepared for their caregiving role. As a result, their quality of life may improve and they may be better able to contribute to stroke survivor recovery, rehabilitation, community re-integration, and quality of life. Given that best practice guidelines [69] recommend the timely education and support of patients and caregivers, it is important to test interventions that can begin to address these recommendations. This study will be the first to examine the timing of caregiver support in a randomized controlled trial. Results from this study will contribute to our understanding of how to implement best practice and meet the changing needs of stroke families as stroke survivors’ transition across care environments.

## Competing interests

The authors declare that they have no competing interests.

## Authors’ contributions

JIC contributed to study conception and design, drafted the manuscript, and approved the final version. GN, MG, MB, GW, and TG contributed to study design, critically reviewed the manuscript, and approved the final version. MH, FS, SP, and AMC contributed to study design and approved the final manuscript. All authors read and approved the final manuscript.

## Pre-publication history

The pre-publication history for this paper can be accessed here:

http://www.biomedcentral.com/1472-6963/14/18/prepub
